# Asteraceae species as potential environmental factors of allergy

**DOI:** 10.1007/s11356-019-04146-w

**Published:** 2019-01-21

**Authors:** Marta Denisow-Pietrzyk, Łukasz Pietrzyk, Bożena Denisow

**Affiliations:** 1 Department of Dermatology, 1st Military Clinical Hospital with the Outpatient Clinic in Lublin, Lublin, Poland; 2 Department of Didactics and Medical Simulation, Chair of Anatomy, Medical University of Lublin, Jaczewskiego 4, 20-094 Lublin, Poland; 3 Department of General, Oncological and Minimally Invasive Surgery, 1st Military Clinical Hospital with the Outpatient Clinic in Lublin, Lublin, Poland; 4 Department of Botany, Subdepartment of Plants Biology, University of Life Sciences in Lublin, Akademicka 15, 20-950 Lublin, Poland

**Keywords:** Allergic contact dermatitis, Irritant contact dermatitis, Allergy, Environment, Asteraceae, Sesquiterpene lactones

## Abstract

The statistics from Europe and the USA have proven a high risk for skin diseases associated with plant contact. Therefore, plant-induced dermatitis is of increasing attention in dermatology. The focus of this paper was to present the current knowledge on aspects of contact allergy related to Asteraceae (Compositae) species. The Asteraceae family is one of the largest in the world with members across all continents. The PubMed/Medline databases have been searched. The Asteraceae representatives consist of diverse secondary metabolites, which exhibit various advantageous effects in humans. In particular, sesquiterpene lactones (SLs) may cause sensitization resulting in skin irritation and inflammation. In this study, we tried to reveal the allergenic potential of several Asteraceae species. The Asteraceae-related allergy symptoms involve eczema, hay fever, asthma, or even anaphylaxis. Furthermore, the evidence of severe cross-reactivity with food and pollen allergens (PFS) in patients sensitive to Asteraceae allergens have been announced. Further identification and characterization of secondary metabolites and possible allergens in Asteraceae are necessary for the better understanding of Asteraceae-related immune response. The Asteraceae allergy screening panel (the SL mix and the Compositae mix of five plant species) is a promising tool to improve allergy diagnostics and therapy.

## Introduction

The statistics from across the world have proven a high risk for skin diseases associated with plant contact (Crosby 2004; Fonacier et al. 2015). Therefore, plant-induced allergies are of increasing attention in medicine (Paulsen et al. 2017; Rozas-Muñoz et al. 2012). A number of different occupations are at risk of plant toxins and allergens exposure. The highest risk is associated with outdoor workers, e.g., farmers, gardeners, forestry and nursery workers, florists, cooks, housekeepers, and grocery store workers (Poljački et al. 2005; Spiewak 2001). In addition to professional activity, skin exposure to plant-related substances can occur during common outdoor or indoor activity (de Jong et al. 1998). Moreover, the increasing popularity of plant extracts in cosmetics (tonics, soaps, shampoos, creams) and massage or aromatherapy fragrance oils raises the chance of contact with hazardous substances (Thomson and Wilkinson 2000).

Plants, including Asteraceae species may imply contact or systemic allergy (Rozas-Muñoz et al. 2012). The Asteraceae species have been identified to produce numerous secondary metabolites, such as polyphenols, flavonoids, diterpenoids, and sesquiterpene lactones (SLs). These metabolites are dissolved or suspended in the latex sap or placed in specific trichomes found on plant organs, i.e., leaf, stem, flowers, seeds, and fruits (Salapovic et al. 2013; Jachuła et al. 2018b). The main group of chemicals relevant to cause allergies and systemic contact dermatitis are sesquiterpene lactones, i.e., lactones with α-methylene group on the γ-lactone ring (Menz and Winkelmann 1987; Nemery and Demedts 1989; Paulsen 2017; Paulsen et al. 2001). In Asteraceae, about 3000 compounds that belong to diverse classes of sesquiterpenoids: guaianolides, eudesmanolides, germacranolides, and pseudoguaianolides have been recognized (Paulsen et al. 2017; Salapovic et al. 2013; Zidorn 2008).

Almost 50% of SLs are potential contact allergens (Menz and Winkelmann 1987). These metabolites are present both in fresh and dried plants in various proportions from 0.01 to 8% per dry weight (Gordon 1999; Neerman 2003). It is also supposed that individuals with contact dermatitis to Asteraceae SLs can react to many other non-Asteraceae SLs-containing plants (Green and Ferguson 1994; Fuchs et al. 2011).

The main objective of this article was to review the evidence for the types of allergic reactions after contact with representatives of the Asteraceae family, which is one of the largest group of flowering plants distributed worldwide. The emphasis was put on the potential allergens from the common species used as popular food, ornamental plants, medicinal plants, and weeds. In particular, the common clinical symptoms of contact and systemic contact dermatitis caused by diverse bioactive molecules present in Asteraceae have been described.

## Methods

The PubMed/Medline databases were searched, from inception to February 2018, using various combination of the following keywords: Asteraceae, Compositae, the names of plant species, sesquiterpene lactones, SLs, and contact dermatitis and related terms: irritant contact dermatitis, allergic contact dermatitis, and systemic contact dermatitis. Each reference retrieved was screened independently by two reviewers (MDP and ŁP), following predefined criteria to determine eligibility for the review.

### Contact and systemic contact (allergic) dermatitis

Contact dermatitis is an inflammatory skin condition accounting for 70–90% of all occupational skin diseases (Adisesh et al. 2013). Contact dermatitis is induced by exposure to an external irritant or allergen and therefore, two types of contact dermatitis: irritant and allergic are distinguished (Rashid and Shim 2016). Approximately 80% of contact dermatitis are irritant contact dermatitis (ICD), which is a non-immunologic response to the direct damage of the skin, by chemical or physical agents (Fonacier and Sher 2014; Pigatto 2015; Tan et al. 2014). The clinical appearances differ between the acute and chronic ICD. The acute ICD includes macules and papules, erythematous, erythemoto-edematous or erytemato-squamous plaques. In the chronic ICD, dry skin, erytemato-squamous dermatitis, hyperkeratosis, and disappearance of fingerprints are found (Nosbaum et al. 2009). The rate of reactions and the severity of changes in skin depend on (i) nature and concentration of the responsible factor; (ii) duration, area, and frequency of contact with an agent; (iii) environment; (iv) skin type; and condition (Slodownik et al. 2008). The mechanism of skin irritation starts with skin damage and is followed by the release of numerous proinflammatory cytokines and chemokines (de Jongh et al. 2007). The primary source of ICD mediators are keratinocytes; however, new insight is given to the mast cells, macrophages, dendritic cells, and natural killers cells (Norman et al. 2008; Vocanson et al. 2005). The cytokines secreted in the ICD are IL-1, IL-6, IL-8, and TNF-α (Nosbaum et al. 2009; Vocanson et al. 2007).

Allergic contact dermatitis (ACD) compromises 20% of cases of contact dermatitis and includes two phases: (i) sensitization— maturation of potential to develop a cutaneous allergic reaction to allergen and (ii) elicitation—skin inflammation developed as a result of repeated exposure to the allergen in a sensitized person (Fonacier and Sher 2014). ACD is a type IV delayed hypersensitivity reaction to an external allergen with the circulating memory T cells involved as the main players. T cells home into the skin during r-exposure to an allergen and activate immunologic reaction causing skin inflammation, usually within 48 h (Burkemper 2015). The activated T cells produce cytokines, e.g., IL-2, IL-17, and INF-α, which further activate and damage skin cells (McFadden et al. 2013). The cellular apoptosis induces inflammation, recruitment, and mobilization of new cells in the skin resulting in eczema (Cavani 2008; Vocanson et al. 2007). The clinical symptoms and signs of ACD consists of erythema, edema, and oozing in the acute phase, while the chronic phase is characterized by lichenified, fissured, and pigmented skin. The location of clinical signs in the ACD is usually limited to the site of contact; however, in contrary to the ICD skin lesions might spread locally or at a distance (Asano et al. 2009; Nicolas et al. 2008). Summary and differential diagnosis between ICD and ACD are presented in Table 1.

**Table 1:** Summary and differential diagnosis between ICD and ACD

Criteria	Irritant contact dermatitis	Allergic contact dermatitis
Risk group	Anyone, especially people with repeated exposure	Previously sensitized, people genetically predisposed
Mechanism	Non-immunologic response to the direct damage of the skin	Immunologic, type IV delayed hypersensitivity reaction
Concentration of factor or allergen	Usually high, positive correlation between power of the agent’s concentration and sin lesion	Might be low, required threshold concentration
Symptoms	Burning, prickling, stinging	Pruritus, erythema, edema
Skin lesions’ area	Limited to the place of irritation	Site of contact, lesions might spread locally or at a distance
Onset of lesions	Appear rapidly, within minutes to hours	Appear within 24–72 h, possible late onset at 7 days after exposure
Diagnostic methods	None	Patch test

Systemic contact (allergic) dermatitis (SCD) is an inflammatory skin disease and occurs in sensitized person after oral, inhalation, intravesical, intravenous, or transcutaneous exposure to the haptens (Nicolas et al. 2008; Veien 2011). Systemic reactions are induced by both humoral and cell-mediated mechanism including T cells and cytokine secretion (Paulsen 2017). Clinical symptoms include local allergic manifestations; however, in a person exposed to allergen, systematically noncutaneous symptoms might develop such as fever, chest pain, and urticarial (Andrews and Scheinman 2011).

Skin severe reactions caused by bioactive chemicals from the representatives of the Asteraceae family have been described worldwide (in North America, Europe, Asia, Australia); however, patient sensitivity varied between geographical regions or seasons and is associated with both sex and age (Gordon 1999; Thomson and Wilkinson 2000). In Europe, Asteraceae-related allergy is among the top ten contact sensitivities, in most cases noted in Central and South Europe (Alexander et al. 2013; Paulsen and Andersen 2016). The allergic reactions after contact with Asteraceae SLs differ between countries, ranging from 0.1% to 2.7%, with a mean prevalence of 1.5% (Paulsen 2017).

Some authors even indicate that the detection for Asteraceae-related skin dermatitis is insufficient due to the low awareness of the problem among patients and their doctors (Spiewak 2001). Typical routes of accidental exposure are skin or eye contact or ingestion (Gordon 1999; Jovanović and Poljacki 2003; Neerman 2003). Positive reactions to Asteraceae allergens (SLs, flavonoids, proteins) may be caused not only by plant allergy, but also by cross-reactivity with, for instance, fragrance terpenes (Paulsen 2017; Paulsen and Andersen 2016).

**Fig. 1:**
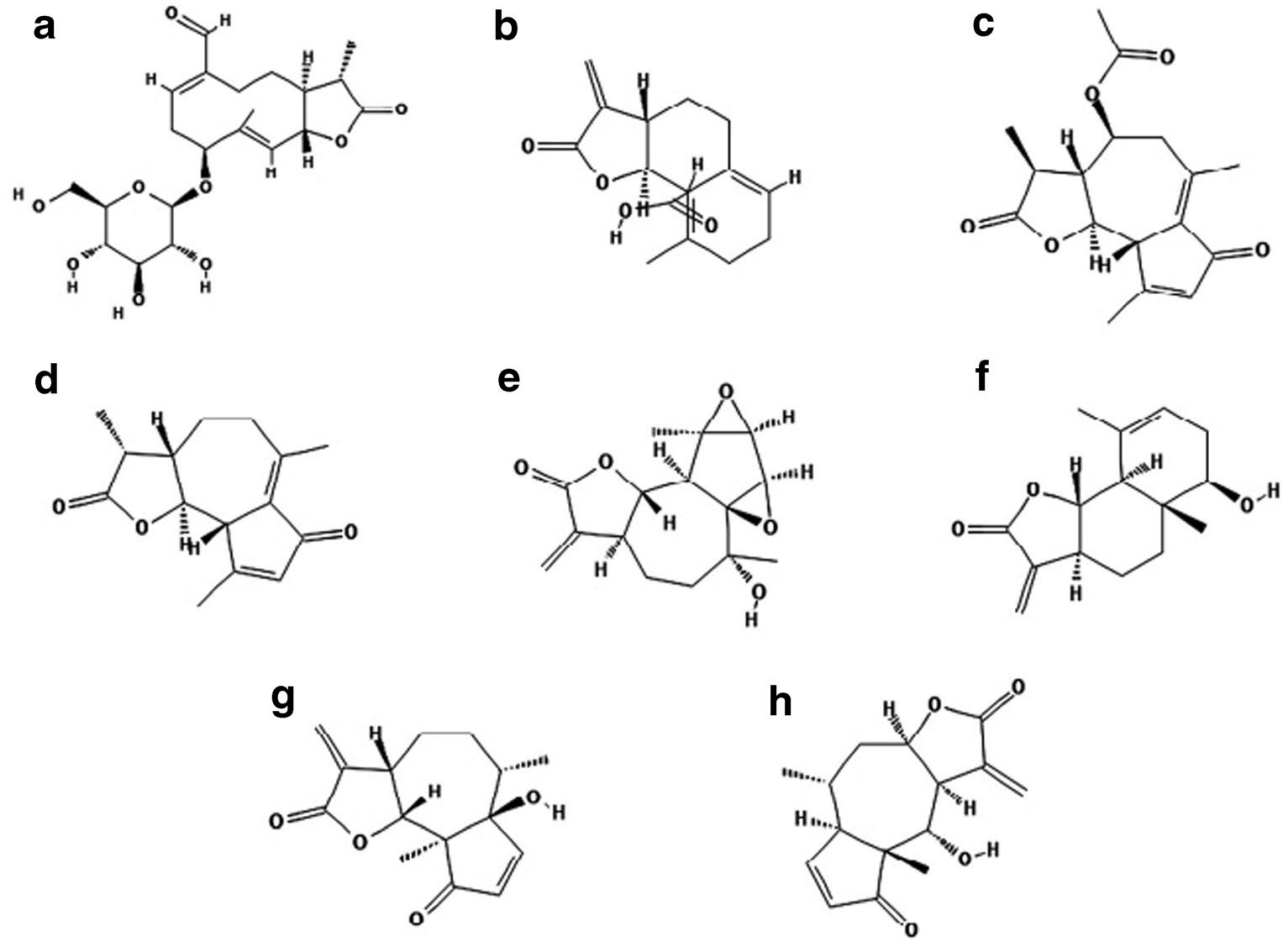
Structures of several common SL compounds found in plant tissue of the Asteraceae (Compositae) species presented according to their chemical classification. 1. Germacranolides—lactuside A (A), taraxinix acid (B); 2. Guaianolides—matricarin (C), achillin (D); 3. Eudesmanolides—artecanin (E), balchanin = santamarin (F); 4. Pseudoguaianolides—parthenin (G), helenalin (H)

Diverse reactions have been documented after transcutaneous absorption of toxins from Asteraceae (Paulsen and Andersen 2016; Zidorn 2008). Generalized eczema (20–30%), eczema of exposed body surfaces, i.e., hands and face (24%), facial eczema (11–28%), eczema of V of the neck, and forearms are found among clinical manifestations of Asteraceae-derivative symptoms (Jovanović and Poljacki 2003). In addition, areas protected from the sunlight exposure such as retroauricular regions (Wilkinson triangle), eyelids, and nasolabial folds are also at risk, allowing its differentiation from a true photo-related dermatitis (Gordon 1999). Among Asteraceae-sensitive individuals, 78.8% exhibit different contact skin inflammations, e.g., to nickel in 33.3% of patients or photosensitivity in 22–75% of persons (Jovanović and Poljacki 2003). Asteraceae allergy screening panel developed by the North American Contact Dermatitis Group comprises of two standard Asteraceae allergens responsible for contact skin inflammation (1) sesquiterpene lactones mix (SL mix; mix of three common SLs (alantolactone, dehydrocostus lactone, and costunolide) and (2) Compositae mix (CM) comprises the biological substances from five Asteraceae species, i.e., *Arnica montana*, *Matriacaria recutica*, *Tanacetum parthenium*, *Tanacetum vulgare*, and *Achillea millefolium* (Alexander et al. 2013). Both the SL mix and the Compositae mix are considered as efficient screening for Asteraceae-related allergy (Green and Ferguson 1994; Paulsen et al. 2001). Potential allergens derivative from Asteraceae species are displayed in Table 2 and structures of several common SLs compounds are shown in Fig. 1.

**Table 2:** Species of the Asteraceae (Compositae) family with potential allergens

Name	Distribution	Allergens
Edible plants		
Lettuce, *Lactuca sativa* L.	Cultivated worldwide	SLs—guaianolides, lactucin, lactucopicrin, 8-deoxylactucopicrin
Endivie, *Cichorium endivia* L.	Mediterranean region	SLs—lactucopicrin, kaempferol malonyl glucoside
Chicory, *Cichorium intybus* var. *sativum* L.	Europe and North America	SLs—grosheimin, guaianolide ixerisoside D, glycosides
Globe artichoke, *Cynara scolymus* L.	Mediterranean region of Europe and North Africa	SLs, flavonoids, hydroxycinnamic acids, tyrosols, and lignans
Sunflower, *Helianthus annuus* L.	Central America, cultivated in moderate climate zones and semi-arid regions	SLs—niveusin B and argophyllin A and B, diterpene acids, grandifloric acid, ciliaric acid and 17-hydroxy-*ent*-isokaur-15(16)-en-19-oic acid, albumins
Ornamental plants		
Chrysanths, *Chrysanthemum* sp.	Native to Asia and northeastern Europe, known and cultivated worldwide	SLs—guaianolides cumambrin A, dihydrocumambrin A, pyrethrum
Dahlia, *Dahlia* sp.	Cultivated in Europe, Asia	SLs—causesin
Zinnia*, Zinnia* sp.	Cultivated in Europe, Asia	SLs—zinagrandinolides A-C (1–3), delta-elemanolide 4
Herbs		
Dandelion, *Taraxacum officinale* L.	Native to Europe and Asia; naturalized worldwide	Taraxinic acid-1ƒ-*O*-*b*--glucopyranoside
Marigold, *Calendula officinalis* L.	Southern Europe; naturalized in temperate regions	Triterpenoids, flavonoids, coumarins, quinones, volatile oil, carotenoids and amino acids
Wild chamomile, *Matricaria chamomilla* L.; *Chamomilla recutita*; Dog fennel, *Anthemis cotula* L.	Common in Europe, North Africa, and Asia	SLs, *a*-peroxyachifolid, herniarin, nobilin, bisabolol coumarin, anthecotulide flavonoids
Echinacea, *Echinacea purpurea* (L.) Moench.)	Native to North America, used worldwide	SLs cimifugaside, caryophyllene
Tansy, *Tanacetum vulgare* L.	Native to Europe and Asia; naturalized in North America	SLs tatridin A,
Feverfew, *Tanacetum parthenium* L.	Common in Europe, North America, Canada	SLs— germacranolides (e.g., parthenolide), eudesmanolides guaianolides, artecanin, artemorin, balchanin, canin, costunolide
Mugwort, *Artemisia vulgaris* L.	Native to Europe, Asia, Northern Africa; naturalized in North America	SLs, psilostachyin, psilostachyin-C, artemisin
Yarrow, *Achillea millefolium* L.	Common in Europe, Asia, and North America	SLs—*a*-peroxyachifolid, flavonoids
Great burdock, *Arctium lappa* L.	Common in Europe, Asia, and North America	SLs, actiopicrin
Arnica, *Arnica montana* L.	Central Europe	SLs—xanthalongin, helenalin
Ragweed, *Ambrosia artemisifolia* L.	Native to North America and Canada; naturalized in Europe	SLs—psilostachyin, psilostachyin B and psilostachyin C; pseudoguaianolides cumanin, peruvin and dihydrocumanin
Santa Maria feverfew, *Parthenium hysterophorus* L.	Native to tropical regions of America; invasive in India, Australia, and Africa	SLs—parthenin

### Asteraceae family—characteristic and representatives

The Asteraceae family (Compositae) is one of the major group of flowering plants, comprising approximately 1000 genera and 23,000 species widespread worldwide (Heywood 1993; Tutin et al. 1980; Czarnecka and Denisow 2014). The Asteraceae species are distributed from the polar zone to the subtropical and tropical zones, except Antarctica (Stevens 2001). The Asteraceae species occur in various habitats and represent 8–20% of native floras (Tutin et al. 1980). The characteristic feature is flower reduction and organizing in the capitulum-type inflorescence. The members are mainly herbaceous species; however, trees and shrubs are also found. Numerous of these species are economically important and are cultivated as crops (Heywood 1993). The plants are grown as vegetables: lettuce (*Lactuca sativa* L.), endive (*Cichorium endivia* L.), cardoon (*Cynara cardunulus* L. var. *sylvestris*), globe artichoke (*Cynara cardunculus* L. var. *scolymus*), common salsify (*Tragopogon porrifolius* L.), black salsify (*Scorzonera hispanica* L.), the garden ornamentals useful in flowering arrangements (e.g., *Ageratum*, *Aster*, *Dahlia*, *Tagetes*—marigold, *Zinnia*); several species are popular as indoor plants (*Chrysanthemum*, *Gerbera*, *Senecio*) (Tutin et al. 1980). A large number of species are recognized as weeds, e.g., *Cirsium* sp., *Carduus* sp., coltsfoot (*Tussilago farfafa* L.), dandelion (*Taraxacum officinale* L.), yarrow (*A. millefolium* L.), tansy (*T. vulgare* L.), and are characteristic for areas with different levels of anthropopressure (Denisow 2011; Wrzesień et al. 2016a, b; Jachuła et al. 2018a, b). Some species are popular as medical herbs, i.e., arnica (*A. montana* L.), German chamomile (*Chamomilla recutita* (L.) Rauch.) or marigold (*Calendula officinalis* L.) (Reider et al. 2001). Invasive plant species are also common among Asteraceae species (Denisow et al. 2017). Some anemophilous Asteraceae species are responsible for severe pollinosis, e.g., common ragweed (*Ambrosia artemisifolia* L.), mugwort (*Artemisia vulgaris* L.), marsh elder (*Iva xanthifolia* Nutt.), or cocklebur (*Xanthium strumarium* L.) (D'Amato et al. 2007).

### Edible Asteraceae species

Lettuce (*L. sativa* L.) is an important leaf vegetable with numerous varieties commonly cultivated worldwide and increasing production in European countries (Paulsen et al. 2001). Among SLs are the guaianolides, of which lactucin, lactucopicrin, and 8-deoxylactucopicrin are the most representative (Salapovic et al. 2013). However, a 9-kDa lipid transfer protein (Lac s 1) is considered the major allergen in lettuce (Hartz et al. 2007). Two Lac s 1 isoforms were recognized, with an amino-acid showing the high sequence identity to Pru p 3 from peach, apple allergen Mal d 3, and to the London plane tree pollen (LTPs pla a 3) (Franck et al. 2000). The allergic patients’ sera showed specific IgE binding to an nLac s protein from the lettuce extract (Hartz et al. 2007). Lettuce protein (Lac s 1) may result in cross-reactivity with other lipid transfer protein-containing foods (LTPs), e.g., from Rosaceae family (peach, apple, apricot); other plant sources (mugwort, peanut, hazelnut, chestnut, grapes, maize, beans, orange, onion, tomatoes, strawberry); pollen; or animal food (milk, fish, sea food, chicken) (Avila Castanon et al. 2002; Diaz et al. 2013; Díaz-Perales et al. 2000; Pastorello et al. 2000; Vila et al. 1998).

In central and northern Europe, the allergy to lettuce is not frequently noted (de Jongh et al. 2007). However, allergy symptoms of a lettuce allergy reaction can invoke after ingestion and can range from mild to severe (Diaz et al. 2013; Vila et al. 1998). In some regions, the species is included among major Asteraceae skin irritants (Alexander et al. 2013). In particular, the allergic response to lettuce is associated with birch pollinosis, and the symptoms are usually limited to the oropharyngeal system (Díaz-Perales et al. 2000). In the Mediterranean countries, the allergic reactions are independent on pollinosis, and the individuals manifest systemic reactions associated with nonspecific lipid transfer proteins (nsLTP) (San Miguel-Moncín et al. 2003). Considering common lettuce consumption, the contact allergy is relatively rarely reported; however, several occupational cases have been documented, therefore, the lettuce-related allergy may be underdiagnosed (Paulsen and Andersen 2016; Vila et al. 1998). In clinical practice, diverse symptoms associated with allergy to lettuce protein have been described, i.e., urticaria, gastrointestinal symptoms, OAS, and angioedema (Hartz et al. 2007).

Occupational contact dermatitis has been revealed in employees working with green business (greenhouse workers, gardeners, cookers) (Helander 1984; Krook 1977). Lettuce has been reported to encounter for lip and facial swelling. In isolated cases, an aggravation of pre-existing dermatitis has been recorded (Oliwiecki et al. 1991). Several patients have been described to have anaphylaxis that occurs in response to lettuce (Morita et al. 2007; Olive-Perez and Pineda 2003; San Miguel-Moncín et al. 2003).

Endivie (*C. endivia* L.), a bitter-leafed vegetable, is particularly common in the Mediterranean region. In this region, the endivie is responsible for 20–30% of skin allergies (Alexander et al. 2013). Occupational hand dermatitis has been reported in SL-sensitive patients (Rozas-Muñoz et al. 2012). The patients with severe chronic skin irritation to lettuce (*L. sativa*) can have cross-sensitivity to endivie (Krook 1977).

Chicory (*Cichorium intybus* var. *sativum* L., succory, coffee weed, cornflower, wild chicory) is a species common in the wild in Europe and North America (Heywood 1993; Tutin et al. 1980). It is of substantial economic, culinary, and medicinal potential. The plant is grown for its roots, which are known for the high concentration of inulin, the polysaccharide that is reported to have diverse advantages to the human body, i.e., enhance the immune system and stabilize blood sugars and lipids level (Figueira et al. 2003). IgE-mediated allergy with skin irritant reactions, facial erythema, dyspnea, chronic eczema, as well as severe bronchospastic reactions has been reported after contact with chicory root or leaves (Das et al. 2016). The skin reaction to chicory allergens may be delayed, and the symptoms may occur even 2 years after the first contact to cultivating chicory plants (Morita et al. 2007). The plant can cross-react with birch pollen, and in some individuals with birch pollen allergies, it causes the oral allergy syndrome (Cadot et al. 2003; Willi et al. 2009). In rare cases, the anaphylactic type I allergy to chicory was also reported (Morita et al. 2007; Olive-Perez and Pineda 2003; Willi et al. 2009).

Globe artichoke (*Cynara scolymus* L., syn.; *C. cardunculus* var*. scolymus* L.) is a perennial herb, native to the Mediterranean region of Europe and North Africa, used as a vegetable plant with edible head inflorescence (Heywood 1993). The plant is used in phytomedicine to enhance the kidneys and stimulate bile acid excretion and flow (Ben Salem et al. 2015). The development of occupational rhinitis and bronchial asthma has been reported in vegetable warehouse workers after sensitization to artichoke (Miralles et al. 2003).

Sunflower (*Helianthus annuus* L., common sunflower) is an annual plant, native to Central America (Heywood 1993). It is widely cultivated as an oilseed crop and livestock forage in semi-arid regions (Tutin et al. 1980). The protein allergens with high cross-reactivity (32, 24, 55, and 55 kDa albumins, LTPs Hel a 1, Hel a 2) have been found in sunflower pollen (de la Hoz et al. 1994; International Union of Immunological Societies Allergen Nomenclature). Pollen allergens differ from seed allergens Hel a 3 (Macias et al. 2014). In patients allergic to sunflower proteins, generalized urticaria, angioedema, oral allergy syndrome, and other symptoms were reported (Vandenplas et al. 1998). Although the sunflower seed dust can result in allergic symptoms with serious anaphylaxis, the incidents are very rare (Vandenplas et al. 1998). The safety of sunflower oil ingestion in patients with IgE-mediated hypersensitivity to sunflower seed was reported by Halsey et al. (1986).

### Ornamental Asteraceae species

Chrysanths (*Chrysanthemum* sp.) are native to Asia and northeastern Europe. In Japan, the chrysanthemum is an imperial and national flower (Tutin et al. 1980). In many European countries (Italy, Poland), the ornamental chrysanthemum is restricted to use mainly in cemetery arrangements (Heywood 1993). The first description of a severe skin irritation after contact with the *Chrysanthemum* plants was made by Howe JS in 1887. Currently, the *Chrysanthemum* species and varieties are considered to be a primary sensitizer and principle agent of contact occupational dermatitis in Western Europe (60%) (Alexander et al. 2013). The allergens in chrysanths (mainly SLs) are found in the flowers and leaves, as well as in the hairs (trichomes) developed on all plant parts (Salapovic et al. 2013). The trichomes easily become airborne and can contact nose and eyes mucosa (Menz and Winkelmann 1987). The symptoms and complaints due to the direct contact with the *Chrysanthemum* plant parts can vary from urticaria to allergic rhinoconjunctivitis and asthma (de Jong et al. 1998). The contact dermatitis often begins with fingerstrips and extend to the face and forearms and can occur minutes after contact (Alexander et al. 2013). Cross-sensitization allergy symptoms after contact with several Asteraceae members (e.g., *Matricaria*, *Solidago*) have also been reported (de Jong et al. 1998).

Dahlia (*Dahlia* sp.) is a perennial ornamental plant (Heywood 1993). The causes of dahlia dermatitis have been described in Asian countries (Nandakishore and Pasricha 1994). Sensitization occurs through direct and airborne skin contact. Allergic symptoms are noted mainly in face and hands (Alexander et al. 2013).

### Asteraceae herbaceous plants

Dandelion (*T. officinale* L.) is a herbaceous perennial weed native to Europe and Asia; however, it is naturalized and found on all continents (Heywood 1993; Tutin et al. 1980). The plant is found in abundance in meadows, roadsides, and ruderal places. In several countries, it is recognized as a severe weed in agriculture and gardening; in others, as a beneficial apicultural plant (Denisow 2011). Dandelion contains many pharmacologically active compounds and is used as herbal medicine in Europe, North America, and China (Schutz et al. 2006). Among potential allergens, an 18-kDa Bet v 1 related-protein with high expression in roots and stems has been extracted from dandelion (Xu et al. 2000). The dandelion sensitivity is expected in patient allergic to the pollen of wind-pollinated Asteraceae (e.g., *Ambrosia*, *Artemisia*, *Iva*) as the cross-reactive epitopes have been shown in several Asteraceae members (Paulsen and Andersen 2016; Syhaieva 2006). For example, the ingestion of bee pollen recommended as food supplementation can result in acute allergic reactions (Cohen et al. 1979; Denisow and Denisow-Pietrzyk 2016; Helander 1984). Several studies described a seasonal cutaneous allergy after contact with dandelion (Cohen et al. 1979; Hausen and Schulz 1978; Ingber 2000; Jovanovic et al. 2004; Lovell and Rowan 1991; Poljacki et al. 2005; Thomson and Wilkinson 2000). In the Korean study, the sensitization to dandelion occurred in 8.5% of patients with respiratory allergy (Lee et al. 2007). In an atopic patient with hay fever, even an anaphylactic reaction has been observed after intake of mixed pollen with 15% of dandelion participation (Chivato et al. 1996).

Arnica (*A. montana* L.) is a herbaceous perennial plant widespread in the nutrient-poor siliceous meadows of Central Europe (Tutin et al. 1980). The plant extracts are used in alternative medicine and cosmetic products. The arnica-related allergy is not often and have been detected in approximately 1.13% patients; however, the contact allergy with skin irritation to arnica have been described (Reider et al. 2001; Rudzki and Grzywa 1977). Given that sensitization to arnica cannot be assessed by testing with the Compositae or sesquiterpene mix alone, the authors suggest that these allergies are more common and contribute considerably to the contact dermatitis, and presumably are recognized as general plant/Asteraceae allergy (Neerman 2003; Reider et al. 2001).

Marigold (*C. officinalis* L., pot marigold, ruddles, common marigold, garden marigold, English marigold, or Scottish marigold) is an herbaceous, very aromatic perennial known in folk medicine (Heywood 1993). The species is native to southern Europe; currently, it is naturalized in temperate regions (Tutin et al. 1980). Calendula extract contains triterpenoids, flavonoids, coumarins, quinones, volatile oil, carotenoids, and amino acids, with multiple medical activities, i.e., anti-inflammatory, cytotoxic, hepatoprotective, spasmolytic, and spasmogenic (Muley et al. 2009; Silva et al. 2007). Marigold extracts are common in diverse creams, which shows the protective action in humans with irritant contact dermatitis (ICD) (Fuchs et al. 2011; Fuchs et al. 2005). Although adverse reactions to marigolds are rare, approximately 2.0% of allergic patients reacted to allergens in marigold, contact skin allergies, and severe anaphylaxis have been noted (D'Amato et al. 2007; Wintzen et al. 2003).

Yarrow (*A. millefolium* L.) is an herbaceous perennial native to temperate areas in Europe, Asia, and North America (Heywood 1993). It is commonly found in grasslands, ruderal areas, and open forests and is also frequently cultivated as an ornamental plant. The plant is an ingredient in herbal teas. One patient had a flare-up of dermatitis after drinking tea made from *A. millefolium* (Wrangsö et al. 1990).

Chamomile is a common name of several plant species spread over Europe, North Africa, and Asia (*Matricaria chamomilla* L.—wild chamomile, German chamomile in Poland, Germany, France; *Anthemis nobilis* L.—common chamomile in England, Spanish, Germany; *Anthemis arvensis*—common chamomile in Asores, Iran, Denmark, Ukraine; and *Anthemis cotula* L.—dog’s fennel, May weed, stinking chamomile) (Heywood 1993; Tutin et al. 1980). Chamomile is considered as a medicinal plant containing diverse bio-active molecules, e.g., terpenoids, flavonoids, and volatile oils, contributing to its medicinal usage and is listed on the FDA’s GRAS, commonly recommended as a safe list (Srivastava et al. 2010). Chamomile plays an important role in phytomedicine and is known from its antispasmodic and sedative usefulness. Therapeutic effects of chamomile herbs or flowers have been established against hay fever, inflammation, muscle spasms, disorders in a menstrual cycle, insomnia, ulcers, wounds, gastrointestinal disorders, rheumatic pain, and hemorrhoids (Zidorn 2008). However, in a low percentage of individuals, chamomile can be dangerous and initiate allergic reactions, including contact dermatitis reactions (Budzinski et al. 2000; Pereira et al. 1997; Rodríguez-Serna et al. 1998). The tests conducted by Budzinski et al. (2000) revealed that 3.1% of the patients develop an Asteraceae-related allergic reaction, and of these individuals, 56.5% demonstrated allergy to chamomile. As another example, in hay fever patients with an inflammation of meibomian glands, the chamomile employed as fluid extract exacerbates the inflammation syndromes (Subiza et al. 1990). It is presumable that reported allergic effects may result from contamination of common chamomile herb by *A. cotula*, the species very similar to the other chamomiles, difficult to distinguish, and known for its allergenic properties (Budzinski et al. 2000). The plant is even classified as poison (Toxic plants ASPCA 2014). The cases of severe anaphylactic reaction have been reported in a 38-year-old Caucasian man and in an 8-year-old boy, who ingested chamomile as a herbal tea (Andres et al. 2009; Subiza et al. 1989). The allergen protein, a homolog of Bet v 1 has been identified in chamomile (Reider et al. 2000). These high-weight molecules (23–50 kDa) may presumably induce the cross-reactivity with foods and pollen allergens. However, the subjects sensitive to mugwort seldom reveal allergenic reaction to chamomile. On the contrary, the patients sensitive to chamomile are usually allergic to mugwort (Barrett et al. 2010). Furthermore, the authors suggest that evidence of cross-reactivity with food and pollen allergens is highly probable in subjects sensitized to chamomile (de la Torre Morín et al. 2001; Reider et al. 2000). In particular, establishing general recognition of safety of chamomile products is needed before usage in children; pregnant women; or patients with allergy, kidney and liver diseases.

Echinacea (*Echinacea purpurea* (L.) Moench.)—purple coneflower) is a herbaceous, perennial plant, native to North America commonly used to enhance the immunology system and prevent against cold infections (Barrett et al. 2010; Stevens 2001). Adverse reactions to Echinacea have been documented in Australian patients (Mullins and Heddle 2002). The Echinacea-related symptoms included severe urticaria (hives), swelling, acute asthma attacks, and anaphylaxis.

Tansy (*T. vulgare* L., syn. *C. vulgare* (L.) Bernh.) is a perennial, herbaceous plant native to Europe and Asia, and is naturalized in North America and Canada. Yellow flower heads are flattened. Fresh tansy herb yields between 0.2% and 0.6% volatile oil of highly variable geographically dependent composition with high participation of monoterpene camphor (Keskitalo et al. 2001). The β-thujone, a compound reported to be highly toxic to brain, liver, and kidney tissues is also a well-known ingredient in tansy (Chiasson et al. 2001). The irritant contact dermatitis has been documented after prolonged exposure to tansy (Paulsen and Andersen 2016). Sesquiterpene lactones (SLs) present in Asteraceae species, e.g., pathenolides, are presumably responsible for severe cross-sensitivity between tansy and chrysanthemum (Paulsen et al. 2001). The allergy for tansy herb have been evidenced in 60.6–77.0% of individuals sensitive to Asteraceae (Paulsen 2017). Clinical symptoms cover the face, hands, and/or forearms, and usually occur after irritant contact with the herb in the wild or through the use of cosmetics (Salapovic et al. 2013; Zidorn 2008).

Feverfew (*T. parthenium* (L.) Sch. Bip., syn. *C. parthenium* (L.) Bernch.) is a perennial plant which grows in most of Europe, North America, and Canada (Tutin et al. 1980; Stevens 2001). It has been used in herbal remedies for centuries (Neerman 2003; Zidorn 2008). The skin irritation symptoms (in eyes, face, neck, and scalp) have been documented in a 45- and 25-year-old woman after usage of moisturizers containing feverfew extracts (Neerman 2003). The patch test with the NACDG revealed positive reaction of both patients to sesquiterpene lactone and to Compositae mix. It is thought that both of these eruptions are a result of contact dermatitis from the Asteraceae family (Killoran et al. 2007).

Mugwort (*A. vulgaris* L. felon herb, chrysanthemum weed, or St. John’s herb, common wormwood). This perennial herb is native to Europe, Asia, Northern Africa, and is naturalized to North America (Heywood 1993; Tutin et al. 1980). The genus *Artemisia* includes 57 species in Europe (Stevens 2001). Mugwort is present in urban, suburban, and rural areas. In tradition folk medicine, the herb is used to release abdominal and menstrual pain and rheumatic arthritis, as an antimalarial drug (Liu et al. 2006). Mugwort (*Artemisia*) and ragweed (*Ambrosia*) are indicated among the most involved in pollinosis among Asteraceae species (D'Amato et al. 2007). In the last decade, the pollen of *Artemisia campestris* have been also identified in airborne pollen in Europe (Grewling et al. 2015). As reported by Park (2005), 42.7% of subjects who experienced the allergic rhinitis and asthma develop positive reactions to mugwort on skin prick testing. Mugwort pollen is known to cross-react with some fruits (peach, apple) and vegetables foods belonging to the Brassicaceae family, such as cauliflower, cabbage, or broccoli (Sugita et al. 2016). The allergic irritation dermatitis revealed after contact with the herbal patch with mugwort ingredient has been reported in a 43-year-old atopic Korean man (Haw et al. 2010). However, the exact *Artemisia* species used for the herbal patch was not identified; therefore, the authors suggest the need for further studies to explain whether there are any differences in skin reaction according to divers *Artemisia* species.

Ragweed (*A. artemisifolia* L.) is an annual herb, native to North America and Canada (Heywood 1993). The species became naturalized in Europe, and currently, it is widespread as an invasive species (Stevens 2001). The sensitization rate against ragweed pollen is high among humans and is compared to that of grass pollen and is expected to increase due to plant migration across Europe (Rodinkova et al. 2018; Buters et al. 2015). The major allergen of ragweed is Amb a 1, a member of the pectatelyases that catalyzes the breakdown of pectin (the major plant cellular wall component). Ragweed cross-reacts with mugwort (*A. vulgaris*). Clinical symptoms of ragweed-related allergy involve allergic dermatitis, oral allergy syndromes, allergic rhinoconjunctivitis, and asthma (Buters et al. 2015; Möller et al. 2002). According to these authors, contact with vegetative parts (leaves) of ragweed may induce hands, underarms, and face eczema with papulo-vesicles or chronic hyperkeratotic eczema. Severe cross-reactivity symptoms between Asteraceae allergens and food allergens, e.g., celery-mugwort-spice syndromes, and mugwort-peach, mugwort-chamomile, mugwort-mustard, ragweed-melon-banana have been also reported (Popescu 2015). Pollen from other Asteraceae species recorded in the atmosphere (i.e., *Iva, Xanthium*) are also known to cause allergy (Sikoparija et al. 2017; Rysiak and Czarnecka 2018).

## Conclusion

In conclusion, Asteraceae species are risk factors for a potential contact and systemic allergy. It is advisable to discriminate the Asteraceae species and use of Asteraceae extracts/herbal teas/cosmetics with caution in highly sensitive persons. The symptoms after contact with Asteraceae species vary widely and could be severe in atopic patients. The evidence of cross-reactivity of Asteraceae species with other plants and anaphylactic reactions have been reported. Moreover, cross-reactivity occurs between Asteraceae allergens and food allergens. Therefore, no allergenicity of Asteraceae products needs to be proven for each case. In diagnosis, it is recommended to use patch tests with either additional plant extracts or commercial Compositae mix adjusted to local conditions.
